# Impact of Swept-Source OCT Angiographic Scanning Speeds on the Detection of Choriocapillaris Flow Deficits in Eyes With AMD

**DOI:** 10.1167/tvst.15.7.16

**Published:** 2026-07-13

**Authors:** Sara Beqiri, Jillene Moxam, Hefu Pan, Bhagavath S. Kumar, Alessandro Berni, Mengxi Shen, Omar S. El-Mulki, Gissel Herrera, Omar Badla, Winston Lam, Eduardo Herrera, Niccolò Ascioti, Viet Hoan Le, Omer Trivizki, Robert C. O'Brien, Nadia K. Waheed, Ruikang K. Wang, Giovanni Gregori, Philip J. Rosenfeld

**Affiliations:** 1 Department of Ophthalmology, Bascom Palmer Eye Institute, University of Miami Miller School of Medicine, Miami, FL, USA; 2 University of Florida, College of Medicine, Gainesville, FL, USA; 3 Department of Bioengineering, University of Washington, Seattle, WA, USA; 4 Department of Ophthalmology, IRCCS San Raffaele Scientific Institute, Milan, Italy; 5 Department of Ophthalmology, Tel Aviv Medical Center, University of Tel Aviv, Tel Aviv, Israel; 6 New England Eye Center, Tufts Medical Center, Tufts University School of Medicine, Boston, MA, USA; 7 Department of Ophthalmology, University of Washington, Seattle, WA, USA

**Keywords:** swept-source OCT angiography (SS-OCTA), scanning-speed, age-related macular degeneration (AMD), choriocapillaris flow deficits (CCFDs)

## Abstract

**Purpose:**

The impact of swept-source optical coherence tomography angiography (SS-OCTA) scanning speeds (interscan times) on the quantification of choriocapillaris flow deficit percentages (CCFD%) was studied in eyes with nonexudative age-related macular degeneration.

**Methods:**

We conducted a retrospective review of 30 pairs of scans from 24 age-related macular degeneration subjects enrolled in an ongoing prospective SS-OCTA imaging study. Patients underwent imaging with same day scans at two scanning speeds: 100 kHz and 200 kHz (corresponding with 5.0-ms and 2.5-ms interscan times, respectively). Best practices for CC slab processing and CCFD quantification were implemented, and the compensation level was optimized for each scan. CCFD% measurements were obtained from the 3-mm and 5-mm fovea-centered circles.

**Results:**

The mean CCFD% measurements from the 3-mm fovea-centered circles for the 100-kHz and 200-kHz scans were 14.20% and 17.17% respectively, and 10.77% and 12.86% for the 5-mm circles. An agreement analysis showed a significant bias with mean values of 2.96% and 2.09% for the 3-mm and 5-mm circles. The limits of agreement extended predominantly in the positive direction with values of −1.96% to 7.89% and −0.60% to 4.78% in the 3-mm and 5-mm circles, respectively, that emphasize the systematically higher CCFD% recorded using the 200-kHz pattern. No confidence interval for the bias included zero, supporting the significant systematic bias across measurement strategies.

**Conclusions:**

SS-OCTA scanning speeds have a significant impact on CCFD% quantification, with slower speeds yielding a lower CCFD%, likely due to the detection of slower CC flow, because of the higher associated interscan times.

**Translational Relevance:**

When following and comparing choriocapillaris flow deficit percentage measurements over time, the same scanning speed should be used at each visit.

## Introduction

The choriocapillaris (CC) is a monolayer network of dense lobular terminal choroidal capillaries that lie adjacent to Bruch's membrane (BM) and facilitate metabolic exchange with the retinal pigment epithelium (RPE) and outer retina.^1^ Optical coherence tomography angiography (OCTA) has been widely used to image the CC in healthy eyes and eyes with age-related macular degeneration (AMD).^2^^–^^17^ The gradual loss of the macular CC perfusion reported in normal aging has been observed histologically and on OCTA imaging.^8^^,^^18^^–^^20^ The major advantage of OCTA imaging is its ability to noninvasively visualize blood flow within the CC in living subjects, and it provides an indirect measurement of CC loss by identifying the extent of detectable CC perfusion as defined by the appearance of CC flow deficits (FDs). These deficits are measured by the absence of detectable CC flow within a defined area, within which the blood flow is below a threshold that would vary depending on the type of OCTA used, such as spectral-domain OCTA or swept-source (SS)-OCTA, the wavelength of the instrument's laser light, the power of the laser light, and the scanning speed of the instrument.^21^

The detection of CCFDs across research groups also varies due to the diversity of imaging protocols, signal-processing algorithms, and the workflows used to quantify CCFDs.^22^ In the pursuit of a standardized methodology that would promote the repeatability and comparability of results, our team has published two evidence-based guideline reports detailing best practices for CC imaging. The first publication by Chu et al.^23^ emphasized the selection of an anatomically correct CC slab positioned at 4 to 20 µm under the BM to accurately visualize the CC and the use of algorithms for binarization and thresholding that reliably quantify CCFDs. In our second and more recent report by Berni et al.,^24^ we focused on strategies to prevent the appearance of artifacts that create false-positive CCFDs and are prevalent in eyes with AMD. In AMD eyes, disease-related anatomical changes that interfere with the measurement of CCFDs include soft drusen, calcified drusen, and hyperpigmentation, also known as hyper-reflective foci, that cause choroidal hypotransmission defects (hypoTD), as well as and the presence of focal areas of atrophy that cause hypertransmission defects (hyperTDs).^25^^–^^35^ Compensation strategies are needed to mitigate the changes in the choroidal OCT signal that can interfere with the accurate measurement of CCFDs in eyes with AMD.^32^^–^^34^ These same compensation strategies can be used with all OCTA instruments.

SS-OCTA imaging has been used to quantify CCFDs using devices with a wide range of scanning speeds from 100.0 kHz to 3.4 MHz.^2^^,^^36^^–^^38^ Although scanning speeds of greater than 400 kHz are presently achieved only by research instruments, commercially available SS-OCTA instruments provide scanning speeds of 100 kHz, 200 kHz, and 400 kHz. In the present study, we use our best practices for imaging the CC and extend these guidelines to understand how different scanning speeds influence the quantification of CCFDs.

## Methods

Patients diagnosed with AMD were enrolled in an ongoing prospective observational SS-OCTA imaging study at the Bascom Palmer Eye Institute. This study was approved by the University of Miami Miller School of Medicine's Institutional Review Board, and all participants signed an informed consent. The study was conducted in alignment with the Declaration of Helsinki and the Health Insurance Portability and Accountability Act of 1996.

### Participants

AMD subjects enrolled in an ongoing prospective SS-OCTA imaging study from May 2025 to August 2025 were selected for this investigation. Eyes with pre-existing exudative macular neovascularization, as well as those with other retinal diseases such as diabetic retinopathy and vascular occlusions were excluded. Additionally, eyes with significant vitreoretinal interface disease that distorted the macular anatomy or with previous vitreoretinal surgery were also excluded.

### Imaging Protocol

All patients underwent fovea-centered 6 × 6 mm SS-OCTA imaging (PLEX Elite 9000; Carl Zeiss Meditec, Dublin, CA) acquired by trained imaging technicians. The PLEX Elite 9000 SS-OCTA instrument has a central wavelength of 1050 nm and dual-speed capability, specifically 100 kHz and 200 kHz, which correspond with 100,000 and 200,000 A-scans per second, producing 500 A-scans per B-scan with a uniform 12-µm spacing both between A-scans and B-scans.

OCTA uses the scattering from moving particles such as red blood cells to visualize the vasculature without requiring a contrast agent. To obtain the angiographic signal, each B-scan is repeated twice at the same location, and the information is processed using the instrument's complex optical microangiographic algorithm.^39^ The time between repeated B-scans is referred to as the interscan time and is the primary factor influencing the OCTA signal. Scanning speed refers to the A-scan frequency, which, in the system used here, determines the interscan time through the acquisition scan protocol. Under otherwise identical settings, images acquired at 100 kHz have an interscan time of approximately 5.0 ms, whereas increasing the A-scan rate to 200 kHz reduces the interscan time to 2.5 ms. Each eye was imaged by the same photographer on the same device during a single session sequentially, using the 100-kHz protocol first, followed by the 200-kHz protocol, to minimize the time between scan protocols.

Multiple scans were acquired using each protocol and these scans were reviewed for image quality and signal strength. Scans were excluded if the signal strength was less than 7 or if significant motion artifacts were present. When several scans were available for an eye on the same date, the highest quality scan was selected.

All scans were reviewed, and eyes with nonexudative macular neovascularization were excluded. The foveal center was localized by identifying the center of the foveal avascular zone on the en face superficial retinal slab, using the device's built-in software. Coordinates were extracted from the intersection of the vertical and horizontal slice navigator lines overlaid on the en face image at that central location.

### Masking and Image Compensation Strategy

The methods used for image processing and CCFD quantification were described previously by Berni et al.^24^ and Beqiri et al.^17^ In summary, we optimized the compensation level for each scan and masked-out regions where the percentage of CCFDs (CCFD%) cannot be measured reliably.

**Figure 1.**
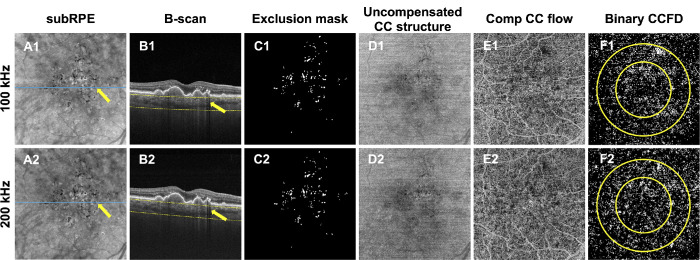
Example of good agreement between CCFD% measurements for the 100-kHz and 200-kHz 6 × 6 mm SS-OCTA scans from an eye with intermediate AMD (iAMD). (**A1**–**F1**, **A2**–**F2**) Workflow and results for the 100-kHz and 200-kHz scans, respectively. (**A1**, **A2**) En face SS-OCTA structural images generated using the sub-RPE slab positioned at 64 to 400 µm under the BM. The *blue line* on (**A1**, **A2**) identifies the position of the B-scans in (**B1**, **B2**), and the *yellow arrow* on (**A1**, **A2**) indicates the position of the hypoTDs that appear as dark foci on the en face sub-RPE image. B-scans from (**B1** and **B2**) confirm the presence of intraretinal hyper-reflective foci that cast shadows identified by the *yellow arrows* and correspond with hypoTDs on the en face images (**A1** and **A2**). The *yellow dashed lines* on (**B1** and **B2**) identify the segmentation boundaries of the sub-RPE slab. (**C1**, **C2**) Total exclusion masks, constructed by identifying all the hypoTDs from the en face sub-RPE image that were confirmed using the corresponding B-scans. All areas represented in *white* by this mask are excluded from CCFD quantification. (**D1**, **D2**) Uncompensated CC structure slabs segmented with boundaries positioned at 4 to 20 µm under the BM. (**E1**, **E2**) Compensated CC flow slabs after an optimal compensation level was applied. (**F1**, **F2**) Binary CCFD maps obtained by applying the fuzzy C-means global thresholding algorithm on the compensated CC flow image. The resulting *white areas* represent the FDs on a black background of detectable flow. The *yellow circles* mark the inner fovea-centered 3-mm circle and the outer fovea-centered 5-mm circle. For the 3-mm circles, the CCFD% for the 100-kHz and 200-kHz scans were 16.6% and 15.8%, respectively. For the 5-mm circles, the CCFD% for the 100-kHz and 200-kHz scans were 14.8% and 15.5%, respectively.

**Figure 2.**
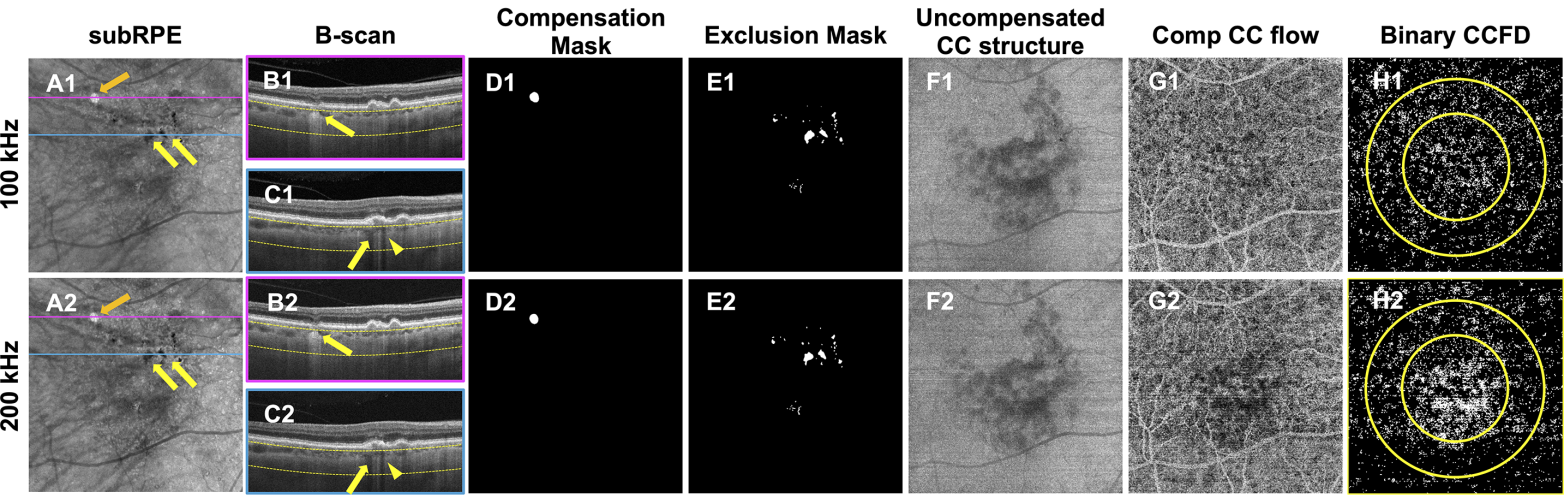
Example of poor agreement between CCFD% measurements for the 100-kHz and 200-kHz 6 × 6 mm SS-OCTA scans from an eye with intermediate AMD (iAMD). (**A1**–**H1** and **A2**–**H2**) Workflow and results for the 100-kHz and 200-kHz scans, respectively. (**A1**, **A2**) En face SS-OCTA structural images generated using the sub-RPE slab positioned at 64 to 400 µm under the BM. The color-coded lines on (**A1** and **A2**) identify the positions of the B-scans in (**B1**–**B2** and **C1**–**C2**). The *orange arrows* on (**A1** and **A2**) indicate the position of a hypertransmission defect (hyperTD) that appears as a bright area on the en face sub-RPE image. B-scans from the *magenta color-coded panels* on (**B1** and **B2**) confirm the presence of RPE and outer retinal attenuation and choroidal hypertransmission identified by a *yellow arrow*. The *yellow arrows* on (**A1** and **A2**) indicate the position of hypoTDs that appear as dark foci on the en face sub-RPE image. B-scans from (**C2**) confirm the presence of a calcified druse and hyper-reflective focus that cast shadows identified by a *yellow arrow* and *yellow arrowhead*, respectively. The *yellow dashed lines* on the B-scans (**B1**–**B2** and **C1**–**C2**) identify the segmentation boundaries of the sub-RPE slab. (**D1**, **D2**) Compensation mask, constructed by identifying areas of hyperTD on the en face sub-RPE image, that were confirmed using the corresponding B-scans. This mask outlines areas that should be avoided by the compensation process, which would artifactually increase CCFD% as described by Berni et al.^24^ However, after the compensation step is complete, these areas are included in the CCFD% quantification. (**E1**, **E2**) Total exclusion mask, constructed by identifying all the hypoTD on the en face sub-RPE image that were confirmed by using the corresponding B-scans, These areas are excluded from the CCFD% quantification. (**F1**, **F2**) Uncompensated CC structure slab segmented with boundaries positioned at 4 to 20 µm under the BM. (**G1**, **G2**) Compensated CC flow slabs after an optimal compensation level was applied. (**H1**, **H2**) Binary CCFD maps obtained by applying the fuzzy C-means global thresholding algorithm on the compensated CC flow image. The resulting *white areas* represent the FDs on a *black background* of detectable flow. The *yellow circles* mark the inner fovea-centered 3-mm circle and the outer fovea-centered 5-mm circle. For the 3-mm circles, the CCFD% for the 100-kHz and 200-kHz scans were 15.3% and 27.4%, respectively. For the 5-mm circles, the CCFD% for the 100-kHz and 200-kHz scans were 12.6% and 16.3%, respectively. These differences between the 100-kHz and 200-kHz protocols for the 3-mm and 5-mm circles exceeds the previously established MDCs of 2% and 1%, respectively, which demonstrates the need to use a consistent protocol throughout a study. The greatest difference is seen in the 3-mm circle matching the location of light attenuation due to drusen shadowing, as shown by the CC structure slabs in (F1 and F2). This may suggest that the reduced signal detected by the 200-kHz scan is misinterpreted as a FD.

Regions that needed to be masked to avoid artifactual decreases in the CCFD% included choroidal hypoTDs, which appear as dark foci on the en face sub-RPE images. These hypoTDs corresponded with areas of decreased light penetration into the choroid due to obstruction of the signal by hyper-reflective foci or calcified drusen, as seen on the corresponding B-scans (Fig. 1, A1, A2, B1, B2 and Fig. 2, A1, A2, C1, C2).^28^^,^^30^^,^^34^^,^^35^ These areas associated with hypoTDs cannot produce reliable CC quantification.

We, therefore, manually outlined these dark foci on the en face sub-RPE image, after confirming their presence using the corresponding B-scan to identify hyper-reflective foci or calcified drusen with an underlying choroidal hypoTD (Fig. 1, C1, C2 and Fig. 2, E1, E2).^32^^,^^33^ Using our custom-built software, we can exclude the regions specified by the exclusion mask from further analysis and quantification.

Choroidal hyperTDs appear as bright foci on the en face sub-RPE images and correspond with areas with increased light penetration into the choroid due to the absence or attenuation of the RPE (Fig. 2, A1, A2, B1, B2).^25^^–^^27^^,^^29^^,^^31^ We define large hyperTDs as bright areas on the en face sub-RPE image that have a greatest linear dimension of 250 µm or greater, are confirmed on corresponding B-scans, and are associated with the loss or attenuation of the outer retina and the RPE in the presence of choroidal hypertransmission. Our recent updated guidelines paper^24^ reported that compensating these regions with hyperTDs leads to artifactually high CCFD% values. Therefore, we manually outlined all large hyperTDs to create a compensation mask that was used to exclude these regions from compensation (Fig. 2, D1, D2). Using our custom-built compensation software, we can avoid compensating over the area specified by this mask. Once compensation is performed, the area is unmasked and included in CCFD% quantification.

A compensation strategy is then implemented to mitigate for light attenuation due to drusen or other RPE irregularities in eyes with AMD. This compensation strategy uses a parameter gamma (γ) that is optimized for each scan to produce the most homogeneous image by finding the lowest standard deviation of pixel illumination across the compensated CC structure slab, which is then used to compensate the CC flow image.^24^

### Segmentation of the CC

We used a semiautomated algorithm to produce a 16-µm-thick CC slab positioned from 4 to 20 µm under the BM. This choice of the CC slab has been validated by previous work and corresponds with the anatomical location of the CC.^23^ The output of the segmentation algorithm was checked and manually corrected within the software wherever necessary. Cases where the segmentation was significantly affected by the presence of nonexudative macular neovascularization, distorted anatomy, and motion or imaging artifacts were excluded.

### CC Signal Thresholding and Binarization

The compensated CC flow image (Fig. 1, E1, E2, and Fig. 2, G1, G2) was thresholded using the fuzzy C-means global thresholding strategy, and perfusion deficits with a size of less than 24 µm, corresponding with a speckle noise smaller than the physiological intercapillary distance, were removed.^2^^–^^4^^,^^8^^,^^16^^,^^23^^,^^40^ A binary CCFD map was produced with white areas representing FDs on a black background of detectable flow. The regions of interest for the comparison of CCFD% measurements were the fovea-centered 3-mm and 5-mm circles (Fig. 1, F1, F2, and Fig. 2, H1, H2).

### Statistical Methods

Hiya et al.^16^ performed a repeatability analysis of CCFD% quantification and established minimal detectable change (MDC) for regions of interest including the 3-mm and 5-mm fovea-centered circles, using the 100-kHz protocol. Differences greater than the MDC in absolute value represent a change outside the normal test–retest variability. For the 3-mm circle, Hiya et al.^16^ reported MDC values of 1.96%, 1.70%, and 1.89% for normal, intermediate AMD, and hyperTD eyes, respectively, and a value of 1.85% across all cases. For the 5-mm circle, the MDC values were 1.36%, 0.85%, and 0.97% for normal, intermediate AMD, and hyperTD eyes, respectively, and 1.08% across all cases. For our analysis, we used rounded values of 2% and 1% as MDCs for the 3-mm and 5-mm circles, respectively, to explore whether using different scanning speeds during a study can lead to the erroneous conclusion that a true change in CCFD% has occurred when it has not.

Agreement between 100-kHz and 200-kHz CCFD% measurements was evaluated with Bland–Altman analysis^41^ and Lin's concordance correlation coefficients^42^ (*r*_c_). Cluster bootstrap resampling was used to account for clustering of fellow eyes. *P* values were obtained via confidence interval inversion and a two-sided *P* value of less than 0.05 was considered statistically significant. All statistical analyses were performed using R version 4.5.1 (The R Foundation for Statistical Computing, Vienna, Austria) with the boot, boot.pval, SimplyAgree, and tidyverse packages.^43^^–^^50^

## Results

### Patient and Eye Characteristics

Thirty eyes of 24 patients with nonexudative AMD were included in this study of CCFD% measurements using two different SS-OCTA scanning speeds. Each eye was imaged at the same visit with both the 100-kHz and 200-kHz protocols. The average time between scan repeats across all eyes was approximately 1 minute. The mean age of the 24 patients was 76.0 ± 10.6 years (range, 57–97 years), and 19 (79.2%) of the patients were female. Of the 30 eyes, 13 had a combination of drusen and hypoTDs and 17 had advanced to large hyperTDs. None of the eyes had nonexudative macular neovascularization.

Figure 1 shows a representative example of an AMD eye with drusen and hypoTDs; no hyperTDs were present. Figure 2 shows a representative example of an AMD eye with both a hyperTD and hypoTDs.

### Group-Level Comparisons of CCFD% Values Between the Two Scanning Speeds

**Figure 3.**
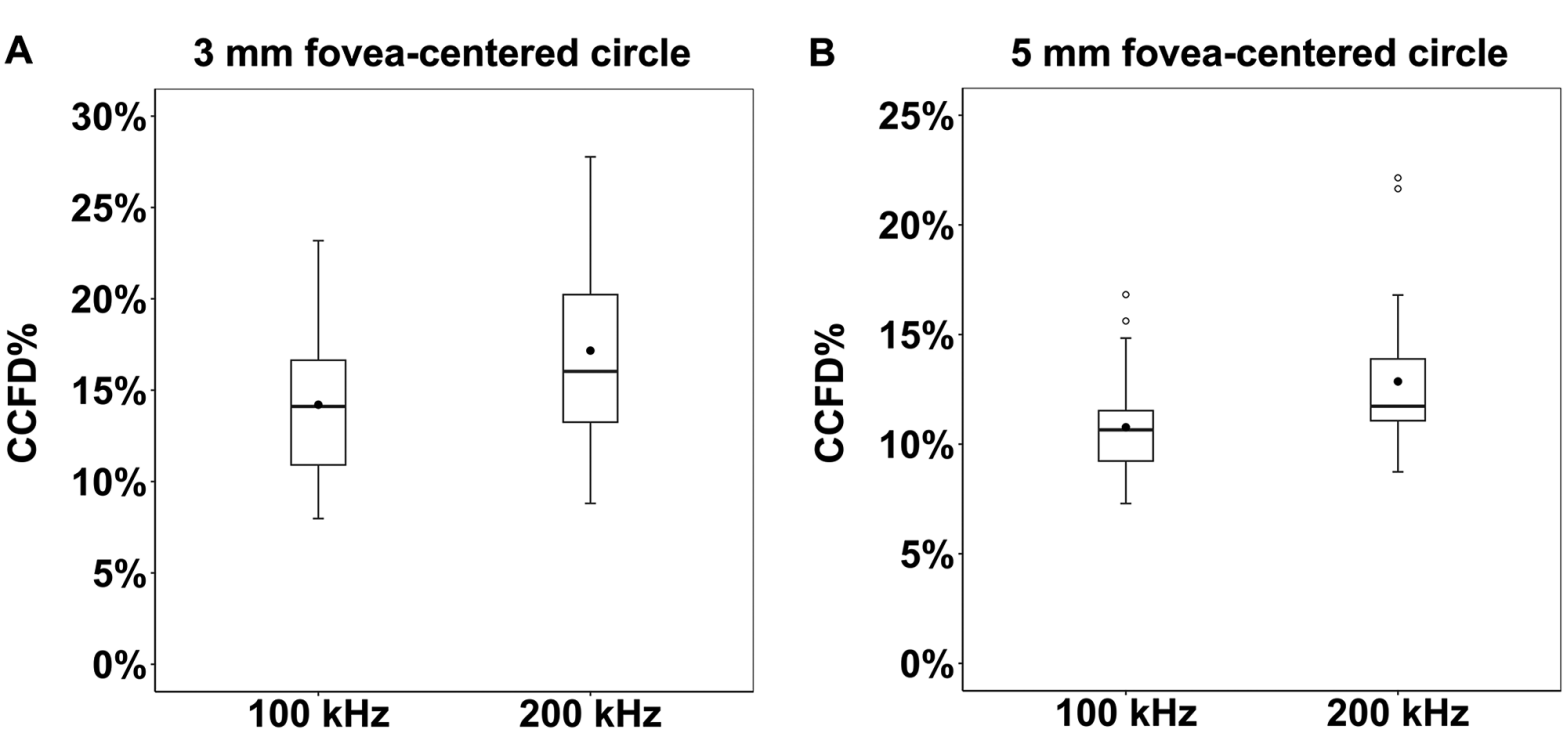
Box-and-whiskers plots of CCFD% measurements from 30 AMD eyes imaged with both the 100-kHz and 200-kHz scanning protocols. The *y*-axis represents the CCFD% values, whereas the *x*-axis represents the 100-kHz and 200-kHz scanning protocols. Each box represents the interquartile range (IQR), the *solid circle* within each box represents the mean, the *horizontal line* represents the median, and the *whiskers* extend to values within ±1.5 × IQR. Outlier points located beyond the whiskers are represented by *circles*. (**A**) CCFD% distributions for the 3-mm circle at 100 kHz and 200 kHz. (**B**) CCFD% distribution for the 5-mm circle at 100 kHz and 200 kHz. Both panels depict a difference in distributions between 100 kHz and 200 kHz, with higher CCFD% results seen in the 200-kHz protocol for the 3-mm and 5-mm circles.

Figure 3 represents a grouped analysis comparing the CCFD% distributions from the two scanning protocols for the two regions of interest—the 3-mm and 5-mm fovea-centered circles—in the 30 eyes. There was a statistically significant difference between the 100-kHz and 200-kHz CCFD% measurements in the 3-mm circles (100 kHz mean CCFD% = 14.20% vs. 200 kHz mean CCFD% = 17.17%; mean difference = 2.96%; *P* < 0.001) and 5-mm circles (100 kHz mean CCFD% = 10.77% vs. 200 kHz mean CCFD% = 12.86%; mean difference = 2.09%; *P* < 0.001) as shown in Figure 3A and Figure 3B, respectively, with the 200-kHz speed providing higher values for each region of interest.

### Eye-Level Comparisons of CCFD% Values Between the Scanning Speeds

**Figure 4.**
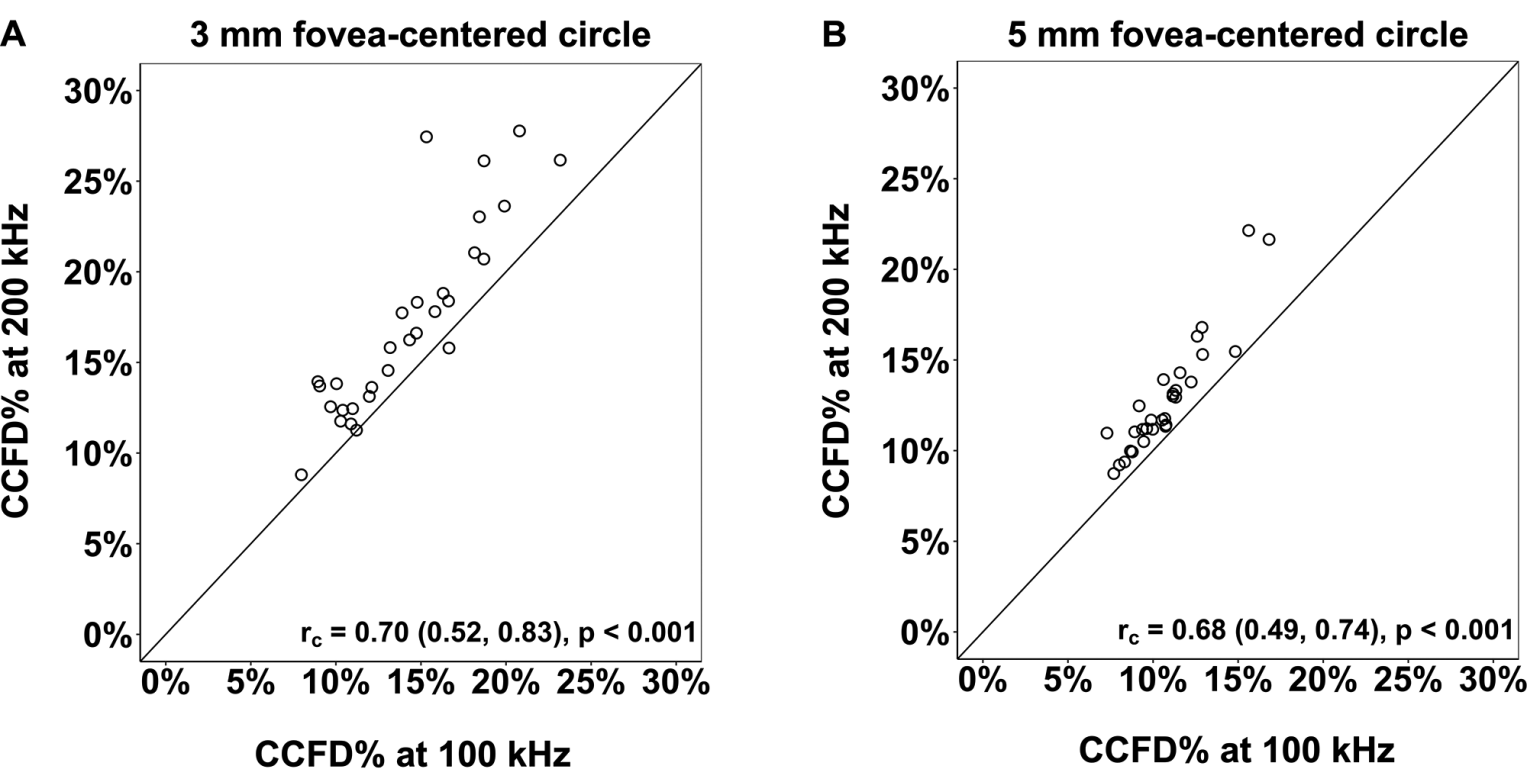
Scatterplot comparisons between CCFD% measurements from 30 AMD eyes, imaged with both the 100-kHz and 200-kHz scanning protocols. Each eye is represented by a datapoint with the *y*-axis representing the CCFD% measurements from the 200-kHz protocol and the *x*-axis representing the CCFD% measurements from the 100-kHz protocol. The *black line* represents the line of equality (slope = 1). (**A**) CCFD% measurements for the 3-mm circle, with all eyes except one, lying above the line of equality. The concordance correlation coefficient *(*r_c_) = 0.70 (95% CI, 0.52 to 0.83; *P* < 0.001), with systematically higher values from the 200-kHz scan. (**B**) CCFD% measurements for the 5-mm circle, with all eyes lying above the line of equality. The concordance correlation coefficient (*r*_c_) = 0.68 (95% CI, 0.49 to 0.74; *P* < 0.001), with systematically higher values from the 200-kHz scan.

Figure 4 represents a scatter plot analysis comparing CCFD% measurements from two scanning speed protocols for the two regions of interest—the 3-mm and 5-mm fovea-centered circles—across 30 eyes, with each dot representing an individual eye.

In the 3-mm circle (Figure 4A), the solid diagonal line represents the line of equality (slope = 1), and all eyes except one display CCFD% values lying above this line, with 16 of them exceeding the 2% MDC. The results derived from the 200-kHz scan are consistently higher with a concordance correlation coefficient (*r*_c_) = 0.70 (95% confidence interval [CI], 0.52–0.83; *P* < 0.001). In the 5-mm circle (Figure 4B), all eyes were above the line of equality and 27 of them exceeded the 1% MDC, showing a similar pattern as the 3-mm region, with a concordance correlation coefficient (*r*_c_) = 0.68 (95% CI, 0.49–0.74; *P* < 0.001).

### Bland–Altman Analysis of CCFD% Values Between the Scanning Speeds

**Figure 5.**
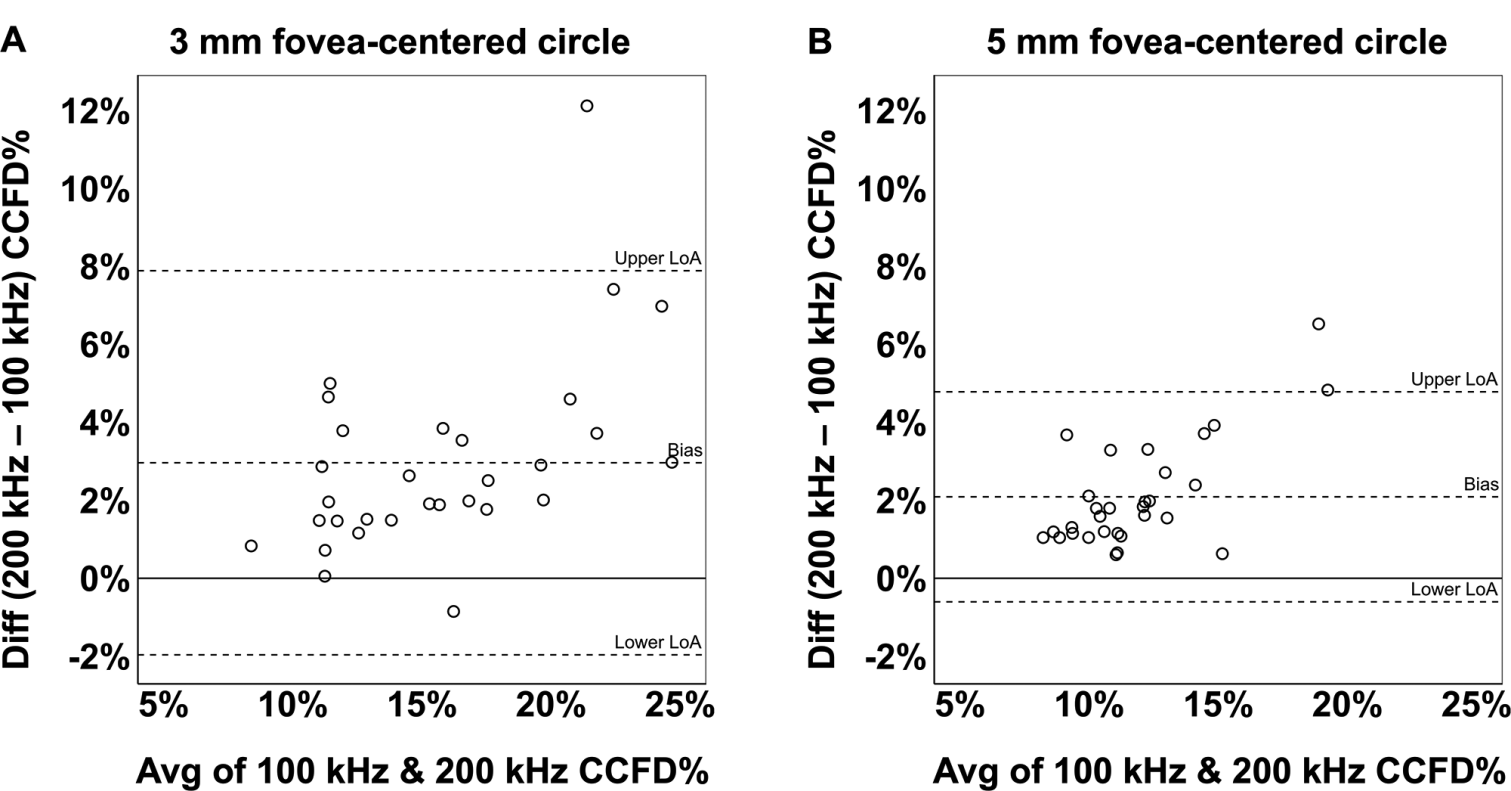
Bland–Altman analysis of agreement between CCFD% measurements from 30 AMD eyes, imaged with both the 100-kHz and 200-kHz scanning protocols. Each eye is represented by a datapoint with the *y*-axis representing the difference between CCFD% measurements from the 200-kHz protocol and 100-kHz protocols, whereas the *x*-axis representing the average of the two measurements. The *middle dashed line* labelled as “bias” represents the average difference between the two scanning protocols. The *upper dotted line* labelled as “upper LoA” represents the upper LoA, which is the highest expected difference between the protocols for 95% of cases and is calculated as Bias + 1.96 ×  SD_differences_. The *lower dashed line* labelled as “lower LoA” represents the lower LoA, which is the lowest expected difference between the protocols for 95% of cases and is calculated as Bias − 1.96 ×  SD_differences._ (**A**) Analysis for the 3-mm fovea-centered circle, showing a bias (mean difference) of 2.96% (95% CI, 2.09%–3.98%), with a lower LoA of −1.96% (95% CI, −3.45% to −0.15%) and an upper LoA of 7.89% (95% CI, 4.88%–10.70%). Differences in CCFD% tended to increase with increasing CCFD% in the 3-mm circle, with greater CCFD% measurements at 200 kHz. (**B**) Analysis for the 5-mm fovea-centered circle, showing a bias (mean difference) of 2.09% (95% CI, 1.57%–2.73%), with a lower LoA of −0.60% (95% CI, −1.17% to 0.25%) and an upper LoA of 4.78% (95% CI, 3.12%–6.29%). Differences in CCFD% tended to increase with increasing CCFD% in the 5-mm circle, with greater CCFD% measurements at 200 kHz, although not as pronounced as in the 3-mm circle. SD, spectral domain.

Figure 5 represents Bland–Altman analyses of agreement between the CCFD% measurements from the two scanning speeds for the two regions of interest—3-mm and 5-mm fovea-centered circles—obtained from the 30 eyes. The results for the 3-mm circle (Figure 5A) show that the Bland–Altman plot has a bias (mean difference) of 2.96% (95% CI, 2.09%–3.98%), with a lower limit of agreement (LoA) of −1.96% (95% CI, −3.45% to −0.15%), and an upper LoA of 7.89% (95% CI, 4.88% to 10.70%). Differences in the CCFD% tended to increase with increasing CCFD% values, with greater CCFD% measurements at 200 kHz.

The Bland–Altman plot in Figure 5B (5-mm circle) shows a bias (mean difference) of 2.09% (95% CI, 1.57% to 2.73%), with a lower LoA of −0.60% (95% CI, −1.17% to 0.25%), and an upper LoA of 4.78% (95% CI, 3.12% to 6.29%). Differences in the CCFD% tended to increase with increasing CCFD% in the 5-mm circle, with greater CCFD% measurements at 200 kHz, although not as pronounced as in the 3-mm circle.

## Discussion

Quantitative imaging of the CC requires attention to each element of the workflow from CC slab selection to masking, compensation, thresholding, registration, and repeatability analyses with the goal of developing a unified and standardized method for measuring the CC using OCTA.^16^^,^^23^^,^^24^ However, the dual-speed capability of several SS-OCTA instruments introduces an additional source of variability, that is, the choice of scanning speed. In this report, we showed that a scanning speed of 200 kHz systemically yielded higher CCFD% measurements than a scanning speed of 100 kHz.

CCFD% values are derived as a binary metric that indicates the presence or absence of detectable CC flow.^4^^,^^9^ Although higher scanning speeds are attractive because they reduce motion artifacts, a protocol change from 100 kHz to 200 kHz, as implemented in this situation, shortens the interscan time between sequential B-scans taken at the same location. This change can complicate the detection of slow CC speeds.^38^ In this study, we acquired same-visit SS-OCTA repeats at both acquisition speeds and compared the CCFD% measurements in the fovea-centered 3-mm and 5-mm circles using the same attention to each element of the workflow. We found that, from a total of 30 eyes, all eyes showed higher values for the 200-kHz scan in the 5-mm circle, with 27 of them exceeding the MDC, and all except one eye showed higher values for the 200-kHz scan in the 3-mm circle, with 16 of them exceeding the MDC.

When grouping all eyes, we demonstrated that there was a statistically significant difference between the 100-kHz and 200-kHz acquisition speeds for both regions. An analysis of agreement between the two protocols showed significant mean bias values of 2.96% for the 3-mm and 2.09% for the 5-mm circles of interest, with no confidence intervals including zero. The LoAs extended predominantly in the positive direction with values of −1.96% to 7.89% and −0.60% to 4.78% in the 3-mm and 5-mm circles, respectively, indicating that the 200-kHz protocol consistently provided higher CCFD% results. These results suggest that a difference in acquisition speeds, with a subsequent reduction in interscan times from approximately 5.0 ms to 2.5 ms, leads to lower measurements of CC perfusion and that changing acquisition speeds during a study is inadvisable.

We should emphasize the 100-kHz and 200-kHz scans for each eye were performed sequentially during the same visit, and the differences observed do not represent disease progression, but rather indicate the effect of the imaging protocol on CCFD% measurements. In OCTA imaging, flow signals are identified by comparing the intensity and phase information from repeated B-scans taken at the same location.^39^ As the interscan time is reduced with faster acquisition speeds, blood flowing at a very slow speed may not be detectable and is instead recorded as a FD. Choi et al.^36^ referred to this phenomenon as the “slowest detectable flow,” whereby any slower flowing blood would be indistinguishable from the background noise and thus undetectable. In contrast, a longer interscan time from a slower acquisition speed such as 100 kHz would allow erythrocytes to progress sufficiently and produce a detectable signal.

Potsaid et al.^51^ explored a range of scanning rates from 100 to 400 kHz for retinal and anterior segment imaging. They found that, when comparing 100 kHz with 200 kHz, the latter showed reduced signal strength and penetration, which could be explained by a combination of shorter time per A-scan and lower optical power per sweep. Our observations from Figure 2, which represented a case with poor agreement, were consistent with these prior findings; we found a significant increase in CCFD% for the 200-kHz scan specifically in the areas of drusen shadowing, which may not have been sufficiently compensated due to lower CC perfusion or due to signal attenuation, resulting in poor signal strength that could not be compensated using our current strategy. Notably, the agreement analyses indicated a higher mean bias (2.96% vs. 2.09%) and wider LoA for the 3-mm region, where typically we expect more drusen to be located, further supporting our hypothesis. In addition, the Bland–Altman plots revealed that differences in the CCFD% between scanning protocols tended to increase with increasing CCFD% values at 200 kHz in both the 3-mm and 5-mm circles. This finding could indicate that a faster scanning speed may be less able to reliably record the CCFD% in an AMD eye with a greater drusen burden. However, because our analysis did not perform a longitudinal intra-eye comparison, we cannot conclude whether the reliability of CCFD% measurements obtained using a faster scanning rate will change over time as the disease progresses.

Key strengths of our work lie in the accurate implementation of best practices for imaging the CC in which we attempted to minimize the confounding effects of possible artifacts on the measurements of CCFD%. We applied the segmentation boundaries for the CC described by Chu et al.,^23^ and manually reviewed each B-scan to ensure correct localization of the CC slab. Additionally, we excluded areas of hypoTD where CC signal cannot be reliably detected, while avoiding compensation in areas of hyperTDs as recommended by Berni et al.^24^ All scans were compensated at their individual optimal level, and a validated thresholding strategy was followed for binarization of the resulting compensated CC flow image.^4^^,^^23^^,^^24^ Furthermore, we compared the results using well-established MDC values from our previous work on the repeatability of the CCFD% quantification.^16^ Our image compensation strategy minimizes the signal attenuation due to drusen and other RPE/BM abnormalities prevalent in AMD eyes. Although we still observed differences in the CCFD% in eyes with drusen, such as in Figure 2, these differences might be even more apparent if compensation had not been applied. Because we have not investigated these scan protocols on uncompensated images, our conclusions apply to images that have been processed according to our previously designed best practices.

A limitation of this study is the size of our population of 30 eyes, which may be considered relatively small. However, our sample represented the spectrum of features seen in dry AMD, consisting of 13 eyes with a combination of drusen, calcified drusen, and hyper-reflective foci, as well as 17 eyes with hyperTDs. This sample size was sufficient to demonstrate a statistically significant difference for both 3-mm and 5-mm fovea-centered circles and the inclusion of more eyes seems unlikely to change these results. Although our cohort does not include normal non-AMD eyes, our previous research clearly showed an increasing CCFD% with advancing age in normal eyes,^8^ and there is no reason to expect the observed trends to be any different than we observed in our AMD population, with the appreciation that the detection of CCFDs in eyes with AMD would be more challenging due to abnormalities in the RPE/BM/CC complex. Thus, we would expect the same 100-kHz vs. 200-kHz differences to be present in both AMD and normal eyes.

Another limitation was the use of a segmentation algorithm that is currently semiautomated, requiring manual oversight and editing where necessary. Additionally, we did not randomize for the order of the imaging protocol. However, the short time difference between consecutive scans minimizes the potential impact of confounding factors such as adaptation in patient fixation or changes in blood pressure.

We recognize that the current quantification of the CCFD% is inherently limited, because it reduces the dynamic CC perfusion into a binary metric. Emerging techniques such as variable interscan time analysis may overcome this limitation by detecting changes in CC flow speeds without reliance on a thresholding strategy; however, this method requires faster scanning speeds of at least 400 kHz.^52^

The absence of a ground truth for in vivo perfusion when reporting the CCFD% limits our ability to establish whether the higher CCFD% obtained with the 200-kHz scans is due to overestimation, or whether the 100-kHz pattern is underestimating the FDs. Nevertheless, in the context of the current approach to CC imaging and quantification, we recommend cautious interpretation of CCFD% results when different acquisition speeds are used. In practice, lower scanning speeds should be prioritized to achieve greater sensitivity for the detection of slower CC speeds. For longitudinal studies, acquisition speeds should remain consistent across visits to enable meaningful comparisons of the CCFD% over time.
